# *N* ^1^,*N* ^4^-Diphenyl-3,6-bis­(phenyl­imino)­cyclo­hexa-1,4-diene-1,4-di­amine

**DOI:** 10.1107/S1600536814002906

**Published:** 2014-02-15

**Authors:** Keiji Ohno, Haruki Maruyama, Takashi Fujihara, Akira Nagasawa

**Affiliations:** a Department of Chemistry, Graduate School of Science and Engineering, Saitama University, Shimo-Okubo 255, Sakura-ku, Saitama 338-8570, Japan; b Comprehensive Analysis Center for Science, Saitama University, Shimo-Okubo 255, Sakura-ku, Saitama 338-8570, Japan

## Abstract

In the title compound, C_30_H_24_N_4_, the central benzo­quinonedi­imine moiety is approximately planar, with a maximum deviation of 0.044 (14) Å. The four terminal phenyl rings are twisted by 44.95 (11), 54.90 (10), 44.98 (10) and 50.68 (11)° with respect to the mean plane the benzo­quinonedi­imine unit. In the crystal, mol­ecules are linked by weak C—H⋯π inter­actions into supra­molecular chains running along the *b*-axis direction.

## Related literature   

For general background to the title compound, see: Kimish (1875 ▶); Rall *et al.* (1998 ▶); Frantz *et al.* (2004 ▶); Siri *et al.* (2005 ▶); Taquet *et al.* (2006 ▶); Schweinfurth *et al.* (2013 ▶); Jeon *et al.* (2013 ▶). For related structures, see: Hughes & Saunders (1956 ▶); Merchant *et al.* (1984 ▶); Siri & Braunstein (2000 ▶); Wenderski *et al.* (2004 ▶); Khramov *et al.* (2006 ▶); Boydston *et al.* (2006 ▶); Huang *et al.*, (2008 ▶); Su *et al.* (2012 ▶).
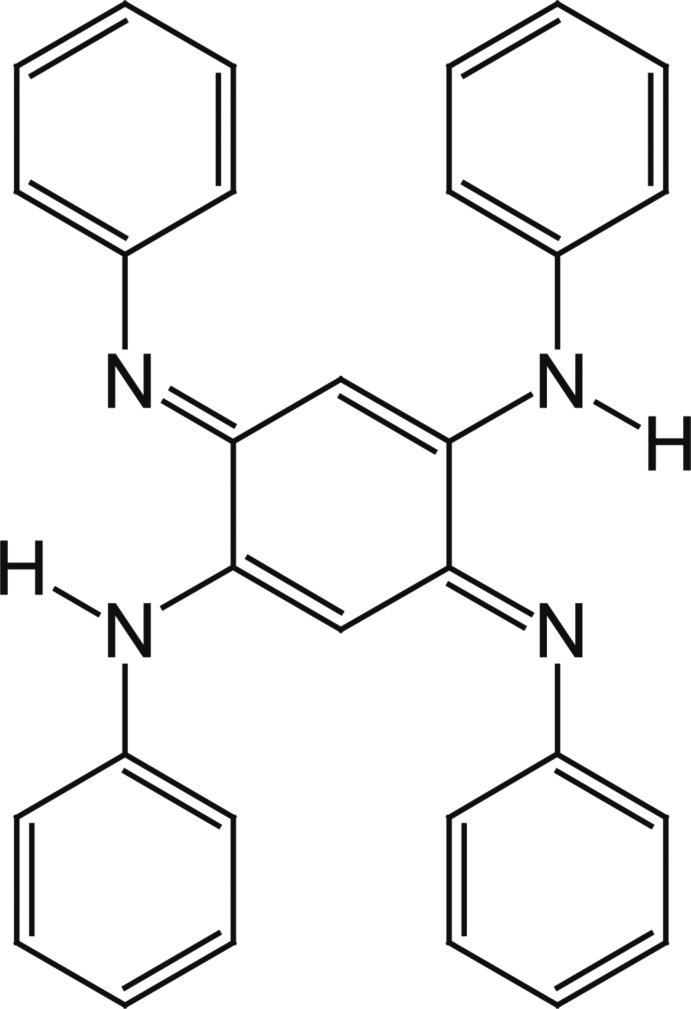


## Experimental   

### 

#### Crystal data  

C_30_H_24_N_4_
*M* *_r_* = 440.53
Triclinic, 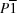

*a* = 8.8858 (12) Å
*b* = 10.0540 (13) Å
*c* = 13.2256 (18) Å
α = 93.343 (3)°
β = 106.760 (3)°
γ = 98.530 (3)°
*V* = 1112.4 (3) Å^3^
*Z* = 2
Mo *K*α radiation
μ = 0.08 mm^−1^
*T* = 173 K
0.30 × 0.20 × 0.10 mm

#### Data collection  

Bruker SMART APEX CCD area-detector diffractometer
Absorption correction: multi-scan (*SADABS*; Bruker, 2001 ▶) *T*_min_ = 0.977, *T*_max_ = 0.992
8166 measured reflections
5291 independent reflections
3273 reflections with *I* > 2σ(*I*)
*R*_int_ = 0.031

#### Refinement  

*R*[*F* ^2^ > 2σ(*F* ^2^)] = 0.064
*wR*(*F* ^2^) = 0.172
*S* = 1.03
5291 reflections
315 parameters
H atoms treated by a mixture of independent and constrained refinement
Δρ_max_ = 0.32 e Å^−3^
Δρ_min_ = −0.24 e Å^−3^

### 

Data collection: *SMART* (Bruker, 2007 ▶); cell refinement: *SAINT* (Bruker, 2007 ▶); data reduction: *SAINT*; program(s) used to solve structure: *SHELXS97* (Sheldrick, 2008 ▶); program(s) used to refine structure: *SHELXL97* (Sheldrick, 2008 ▶); molecular graphics: *SHELXTL* (Sheldrick, 2008 ▶); software used to prepare material for publication: *SHELXTL*.

## Supplementary Material

Crystal structure: contains datablock(s) global, I. DOI: 10.1107/S1600536814002906/xu5766sup1.cif

Structure factors: contains datablock(s) I. DOI: 10.1107/S1600536814002906/xu5766Isup2.hkl

Supporting information file. DOI: 10.1107/S1600536814002906/xu5766Isup3.cml

CCDC reference:

Additional supporting information:  crystallographic information; 3D view; checkCIF report

## Figures and Tables

**Table 1:** Hydrogen-bond geometry (Å, °) *Cg*2 and *Cg*4 are the centroids of the C7–C12 and C19–C24 benzene rings, respectively.

*D*—H⋯*A*	*D*—H	H⋯*A*	*D*⋯*A*	*D*—H⋯*A*
C8—H8⋯*Cg*4^i^	0.95	2.84	3.675 (2)	148
C14—H14⋯*Cg*2^ii^	0.95	2.81	3.673 (3)	151

Symmetry codes: (i) 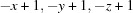; (ii) 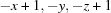.

**Fig. 1.**
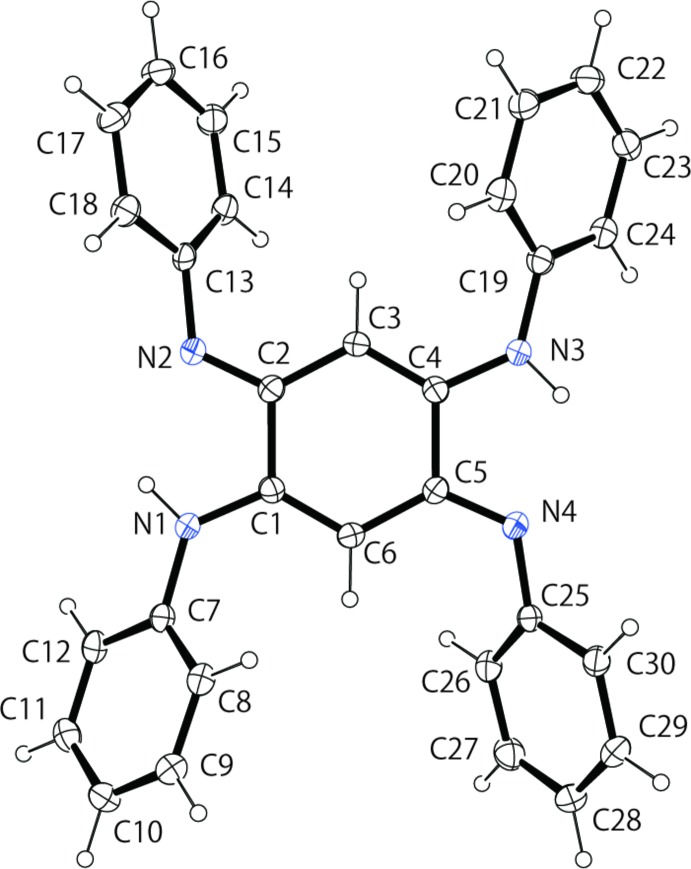
The molecular structure of I, with displacement ellipsoids drawn at the 50% probability level.

## supplementary crystallographic information

### 1. Comment

*N*^1^,*N*^4^-Diphenyl-3,6-bis(phenylimino)cyclohexa-1,4-diene-1,4-diamine (I) was synthesized as early as in 1875 (Kimish, 1875) and called azophenine. Then I and its derivatives were obtained from aniline or substituted anilines in various ways, *e.g.* the oxidation of *N*,*N'*-diphenyl-*p*-phenylenediamine with mercury(II) oxide to give *p*-benzoquinone diamine followed by heating with aniline (Hughes & Saunders, 1956), heating of anilines with dry copper(II) chloride on a steam bath (Merchant *et al.*, 1984), and the Buchwald-Hartwig cross coupling reaction (Wenderski *et al.*, 2004). The molecular structures of the derivatives except I have been investigated (Boydston *et al.*, 2006; Khramov *et al.*, 2006; Huang *et al.*, 2008). This class of compounds forms various metal complexes (Rall *et al.*, 1998; Siri & Braunstein, 2000; Frantz *et al.*, 2004; Siri *et al.*, 2005; Su *et al.*, 2012), some of which exhibit novel properties (Taquet *et al.*, 2006; Schweinfurth *et al.*, 2013), *e.g.* one-electron reduced dinuclear Fe complex with I behaves as a single molecule magnet with the strongest exchange coupling (Jeon *et al.*, 2013).

The crystals of I were obtained in the process of a preparation of V^IV^ complex using aniline and [V^IV^(O)(*η*^2^-ox)(H_2_O)_3_] (ox^2-^ = oxalate) in air. The reaction occurs neither under an argon atmosphere nor when less than four equivalents of aniline to [V^IV^(O)(*η*^2^-ox)(H_2_O)_3_] was used. These results suggest that the reaction proceeds through coordination of aniline to [V^IV^(O)(*η*^2^-ox)(H_2_O)_3_], which may act as a catalyst of oxidation pentamerization of aniline to form I.

The red crystals contain only I, and the structure is in triclinic *P*-1 space group. The atoms of diamino-benzoquinonediimine moiety of I are coplanar. The bond lengths of C(1)–N(1) and C(4)–N(3) correspond to C–N single bond, and that of C(2)–N(2) and C(5)–N(4) correspond to C–N double bond. The bond angles of C(1)—N(1)—C(7) and C(4)—N(3)—C(19) are 128.74 (18) and 127.80 (19)°, respectively, and that of C(2)═N(2)—C(13) and C(5)═N(4)—C(25) are 121.28 (18) and 123.00 (19)°, respectively, indicating that N(1 and 3) and N(2 and 4) are attributed to amine (*sp*^3^) and imine (*sp*^2^) nitrogen atoms, respectively. The bond length of C(2)–C(3) is similar with that of C(5)–C(6), corresponding to that of a single bond, and those of C(3)–C(4) and C(1)–C(6) are similar with each other, corresponding to that of a double bond. The bond lengths of C(1)–C(2) and C(4)–C(5) are slightly longer than the above mentioned single bonds. These results indicate that each of two N–C–C–C–N zigzag shaped moieties shows a bond alternation, but no conjugation between them.

### 2. Experimental

The V^IV^ complex [V^IV^(O)(*η*^2^-ox)(H_2_O)_3_] was purchased as "VO(ox)^.^*n*H_2_O" from Wako Chemicals, and used without further purification. A solution of aniline (27.9 g, 300 mmol) in EtOH (50 cm^3^) was added to a solution of VO(ox)^.^*n*H_2_O (1.13 g, 3.00 mmol) in a mixture of EtOH (50 cm^3^) and H_2_O (100 cm^3^). The reaction mixture was set aside for 2 weeks at room temperature in air. The precipitated crystals were filterd off, washed with H_2_O and EtOH, successively, and dried. Yield 1.34 g. (5.1%). ^1^H NMR / CDCl_3_: *δ* 8.22 (s, 2H, N*H*), 7.41–6.88 (m, 20H, Ph*H*), 6.21 (s, 2H, C*H*). MALDI TOF MS: 441 (*M*+1). UV-vis / CH_2_Cl_2_, *λ*/nm (*ε*/*M*^-1^cm^-1^): 290 (46000), 379 (30000).

### 3. Refinement

The H atoms of NH moieies were located from a Fourier difference map and refined isotrpically. Other H atoms were placed at idealized positions with C—H = 0.95 Å, and refined in ridig mode with *U*_eq_(H) = 1.2*U*_iso_(C).

### Crystal data

**Table:**

C_30_H_24_N_4_	*Z* = 2
*M**_r_* = 440.53	*F*(000) = 464
Triclinic, *P*1	*D*_x_ = 1.315 Mg m^−^^3^
Hall symbol: -P 1	Mo *K*α radiation, λ = 0.71073 Å
*a* = 8.8858 (12) Å	Cell parameters from 1264 reflections
*b* = 10.0540 (13) Å	θ = 2.4–25.9°
*c* = 13.2256 (18) Å	µ = 0.08 mm^−^^1^
α = 93.343 (3)°	*T* = 173 K
β = 106.760 (3)°	Plate, red
γ = 98.530 (3)°	0.30 × 0.20 × 0.10 mm
*V* = 1112.4 (3) Å^3^	

### Data collection

**Table:**

Bruker SMART APEX CCD area-detector diffractometer	5291 independent reflections
Radiation source: fine-focus sealed tube	3273 reflections with *I* > 2σ(*I*)
Graphite monochromator	*R*_int_ = 0.031
Detector resolution: 8.366 pixels mm^-1^	θ_max_ = 28.0°, θ_min_ = 1.6°
φ and ω scans	*h* = −10→11
Absorption correction: multi-scan (*SADABS*; Bruker, 2001)	*k* = −12→13
*T*_min_ = 0.977, *T*_max_ = 0.992	*l* = −17→8
8166 measured reflections	

### Refinement

**Table:**

Refinement on *F*^2^	Primary atom site location: structure-invariant direct methods
Least-squares matrix: full	Secondary atom site location: difference Fourier map
*R*[*F*^2^ > 2σ(*F*^2^)] = 0.064	Hydrogen site location: inferred from neighbouring sites
*wR*(*F*^2^) = 0.172	H atoms treated by a mixture of independent and constrained refinement
*S* = 1.03	*w* = 1/[σ^2^(*F*_o_^2^) + (0.0775*P*)^2^ + 0.0548*P*] where *P* = (*F*_o_^2^ + 2*F*_c_^2^)/3
5291 reflections	(Δ/σ)_max_ < 0.001
315 parameters	Δρ_max_ = 0.32 e Å^−^^3^
0 restraints	Δρ_min_ = −0.24 e Å^−^^3^

### Special details

**Table:**

Geometry. All e.s.d.'s (except the e.s.d. in the dihedral angle between two l.s. planes) are estimated using the full covariance matrix. The cell e.s.d.'s are taken into account individually in the estimation of e.s.d.'s in distances, angles and torsion angles; correlations between e.s.d.'s in cell parameters are only used when they are defined by crystal symmetry. An approximate (isotropic) treatment of cell e.s.d.'s is used for estimating e.s.d.'s involving l.s. planes.Least-squares planes (*x*,*y*,*z* in crystal coordinates) and deviations from them (* indicates atom used to define plane)5.6034 (0.0038) *x* + 5.0416 (0.0058) *y* + 12.9434 (0.0071) *z* = 8.2491 (0.0021)* 0.0006 (0.0008) C10 * -0.0066 (0.0008) C11 * 0.0067 (0.0009) C12 * -0.0008 (0.0009) C13 * -0.0053 (0.0009) C14 * 0.0053 (0.0008) C15Rms deviation of fitted atoms = 0.0049- 7.4963 (0.0028) *x* + 1.7481 (0.0065) *y* + 10.6592 (0.0082) *z* = 4.5261 (0.0067)Angle to previous plane (with approximate e.s.d.) = 86.71 (0.03)* 0.0118 (0.0008) C4 * -0.0134 (0.0008) C5 * 0.0021 (0.0009) C6 * 0.0111 (0.0009) C7 * -0.0128 (0.0009) C8 * 0.0013 (0.0009) C9Rms deviation of fitted atoms = 0.0101- 4.5932 (0.0050) *x* + 6.9089 (0.0064) *y* + 12.6394 (0.0086) *z* = 6.3197 (0.0043)Angle to previous plane (with approximate e.s.d.) = 30.79 (0.06)* 0.0005 (0.0006) C1 * -0.0005 (0.0006) C2 * 0.0005 (0.0006) C3 * -0.0005 (0.0006) C1_$1 * 0.0005 (0.0006) C2_$1 * -0.0005 (0.0006) C3_$1 - 3.3572 (0.0015) C13_$2 - 3.6100 (0.0013) C14_$2 - 3.7830 (0.0014) C15_$2Rms deviation of fitted atoms = 0.0005- 4.5932 (0.0050) *x* + 6.9089 (0.0064) *y* + 12.6394 (0.0086) *z* = 6.3197 (0.0043)Angle to previous plane (with approximate e.s.d.) = 0.00 (0.10)* 0.0005 (0.0006) C1 * -0.0005 (0.0006) C2 * 0.0005 (0.0006) C3 * -0.0005 (0.0006) C1_$1 * 0.0005 (0.0006) C2_$1 * -0.0005 (0.0006) C3_$1 - 3.7228 (0.0017) C10_$2 - 3.4796 (0.0019) C11_$2 - 3.2849 (0.0018) C12_$2Rms deviation of fitted atoms = 0.0005- 5.6034 (0.0038) *x* + 5.0416 (0.0058) *y* + 12.9434 (0.0070) *z* = 2.9266 (0.0041)Angle to previous plane (with approximate e.s.d.) = 10.70 (0.08)* 0.0006 (0.0008) C10_$2 * -0.0066 (0.0008) C11_$2 * 0.0067 (0.0009) C12_$2 * -0.0008 (0.0009) C13_$2 * -0.0053 (0.0009) C14_$2 * 0.0053 (0.0008) C15_$2Rms deviation of fitted atoms = 0.0049- 4.5932 (0.0050) *x* + 6.9089 (0.0064) *y* + 12.6394 (0.0086) *z* = 6.3197 (0.0043)Angle to previous plane (with approximate e.s.d.) = 10.70 (0.08)* 0.0005 (0.0006) C1 * -0.0005 (0.0006) C2 * 0.0005 (0.0006) C3 * -0.0005 (0.0006) C1_$1 * 0.0005 (0.0006) C2_$1 * -0.0005 (0.0006) C3_$1Rms deviation of fitted atoms = 0.0005

### Fractional atomic coordinates and isotropic or equivalent isotropic displacement parameters (Å^2^)

**Table:**

	*x*	*y*	*z*	*U*_iso_*/*U*_eq_	
C1	0.6215 (2)	0.1790 (2)	0.48956 (16)	0.0279 (5)	
C2	0.5987 (2)	0.2013 (2)	0.59667 (16)	0.0272 (5)	
C3	0.4786 (3)	0.2780 (2)	0.60555 (17)	0.0291 (5)	
H3	0.4665	0.2981	0.6736	0.035*	
C4	0.3815 (3)	0.3228 (2)	0.51985 (17)	0.0290 (5)	
C5	0.3993 (2)	0.2946 (2)	0.41226 (16)	0.0272 (5)	
C6	0.5248 (3)	0.2248 (2)	0.40391 (17)	0.0298 (5)	
H6	0.5412	0.2100	0.3366	0.036*	
C7	0.8056 (2)	0.0811 (2)	0.40583 (17)	0.0305 (5)	
C8	0.8318 (3)	0.1764 (2)	0.33796 (17)	0.0340 (5)	
H8	0.8037	0.2632	0.3456	0.041*	
C9	0.8982 (3)	0.1449 (2)	0.26007 (18)	0.0377 (6)	
H9	0.9168	0.2109	0.2144	0.045*	
C10	0.9386 (3)	0.0192 (2)	0.24679 (19)	0.0392 (6)	
H10	0.9830	−0.0019	0.1918	0.047*	
C11	0.9138 (3)	−0.0753 (2)	0.31417 (19)	0.0368 (6)	
H11	0.9405	−0.1624	0.3054	0.044*	
C12	0.8501 (3)	−0.0439 (2)	0.39462 (18)	0.0336 (5)	
H12	0.8368	−0.1084	0.4424	0.040*	
C13	0.6813 (3)	0.1632 (2)	0.77728 (16)	0.0286 (5)	
C14	0.5421 (3)	0.1153 (2)	0.80110 (18)	0.0340 (5)	
H14	0.4491	0.0748	0.7456	0.041*	
C15	0.5379 (3)	0.1259 (2)	0.90426 (19)	0.0400 (6)	
H15	0.4413	0.0938	0.9191	0.048*	
C16	0.6711 (3)	0.1823 (2)	0.98623 (19)	0.0396 (6)	
H16	0.6666	0.1908	1.0572	0.048*	
C17	0.8115 (3)	0.2265 (2)	0.96395 (18)	0.0381 (6)	
H17	0.9048	0.2642	1.0202	0.046*	
C18	0.8180 (3)	0.2163 (2)	0.86100 (17)	0.0339 (5)	
H18	0.9159	0.2457	0.8469	0.041*	
C19	0.2077 (3)	0.4243 (2)	0.60850 (18)	0.0324 (5)	
C20	0.3155 (3)	0.4832 (2)	0.70564 (19)	0.0386 (6)	
H20	0.4267	0.5000	0.7138	0.046*	
C21	0.2614 (3)	0.5167 (2)	0.78905 (19)	0.0399 (6)	
H21	0.3360	0.5538	0.8555	0.048*	
C22	0.1014 (3)	0.4976 (2)	0.77844 (19)	0.0411 (6)	
H22	0.0655	0.5217	0.8369	0.049*	
C23	−0.0066 (3)	0.4433 (2)	0.68263 (19)	0.0373 (6)	
H23	−0.1176	0.4322	0.6745	0.045*	
C24	0.0450 (3)	0.4049 (2)	0.59809 (19)	0.0352 (5)	
H24	−0.0305	0.3651	0.5327	0.042*	
C25	0.2863 (2)	0.3163 (2)	0.22856 (17)	0.0284 (5)	
C26	0.2740 (3)	0.1896 (2)	0.17692 (18)	0.0344 (5)	
H26	0.2778	0.1129	0.2157	0.041*	
C27	0.2564 (3)	0.1740 (3)	0.06994 (19)	0.0426 (6)	
H27	0.2469	0.0863	0.0354	0.051*	
C28	0.2524 (3)	0.2840 (3)	0.01207 (19)	0.0447 (6)	
H28	0.2436	0.2729	−0.0613	0.054*	
C29	0.2612 (3)	0.4105 (3)	0.06249 (19)	0.0401 (6)	
H29	0.2575	0.4867	0.0232	0.048*	
C30	0.2753 (3)	0.4270 (2)	0.16866 (17)	0.0327 (5)	
H30	0.2777	0.5139	0.2019	0.039*	
H1	0.777 (3)	0.075 (3)	0.551 (2)	0.049 (8)*	
H2	0.197 (3)	0.394 (3)	0.453 (2)	0.057 (8)*	
N1	0.7414 (2)	0.1088 (2)	0.48930 (15)	0.0334 (5)	
N2	0.6936 (2)	0.15137 (18)	0.67317 (14)	0.0296 (4)	
N3	0.2589 (2)	0.3886 (2)	0.52080 (15)	0.0361 (5)	
N4	0.2966 (2)	0.34011 (18)	0.33658 (14)	0.0300 (4)	

### Atomic displacement parameters (Å^2^)

**Table:**

	*U*^11^	*U*^22^	*U*^33^	*U*^12^	*U*^13^	*U*^23^
C1	0.0257 (11)	0.0305 (12)	0.0276 (11)	0.0063 (9)	0.0076 (9)	0.0019 (9)
C2	0.0260 (11)	0.0272 (11)	0.0268 (11)	0.0027 (9)	0.0064 (9)	0.0036 (9)
C3	0.0331 (12)	0.0324 (12)	0.0248 (11)	0.0103 (9)	0.0111 (9)	0.0036 (9)
C4	0.0299 (11)	0.0301 (12)	0.0297 (12)	0.0090 (9)	0.0108 (9)	0.0046 (9)
C5	0.0267 (11)	0.0277 (11)	0.0262 (11)	0.0043 (9)	0.0064 (9)	0.0053 (9)
C6	0.0302 (12)	0.0367 (13)	0.0238 (11)	0.0081 (9)	0.0087 (9)	0.0042 (9)
C7	0.0223 (11)	0.0398 (13)	0.0277 (11)	0.0074 (9)	0.0037 (9)	0.0035 (10)
C8	0.0327 (12)	0.0338 (13)	0.0340 (13)	0.0082 (10)	0.0060 (10)	0.0058 (10)
C9	0.0393 (13)	0.0411 (14)	0.0336 (13)	0.0088 (11)	0.0108 (10)	0.0070 (11)
C10	0.0383 (14)	0.0460 (15)	0.0375 (13)	0.0119 (11)	0.0163 (11)	0.0020 (11)
C11	0.0336 (13)	0.0325 (13)	0.0443 (14)	0.0100 (10)	0.0104 (11)	0.0005 (11)
C12	0.0250 (11)	0.0352 (13)	0.0377 (13)	0.0063 (9)	0.0042 (9)	0.0066 (10)
C13	0.0326 (12)	0.0269 (11)	0.0273 (12)	0.0129 (9)	0.0059 (9)	0.0068 (9)
C14	0.0286 (12)	0.0384 (13)	0.0326 (13)	0.0077 (10)	0.0039 (9)	0.0072 (10)
C15	0.0387 (14)	0.0473 (15)	0.0390 (14)	0.0117 (11)	0.0159 (11)	0.0127 (12)
C16	0.0507 (16)	0.0438 (14)	0.0272 (12)	0.0126 (12)	0.0136 (11)	0.0047 (11)
C17	0.0424 (14)	0.0383 (14)	0.0282 (12)	0.0047 (11)	0.0036 (10)	0.0010 (10)
C18	0.0331 (12)	0.0317 (12)	0.0343 (13)	0.0040 (10)	0.0067 (10)	0.0056 (10)
C19	0.0375 (13)	0.0299 (12)	0.0332 (12)	0.0106 (10)	0.0126 (10)	0.0082 (10)
C20	0.0344 (13)	0.0398 (14)	0.0403 (14)	0.0075 (11)	0.0080 (11)	0.0078 (11)
C21	0.0470 (15)	0.0392 (14)	0.0313 (13)	0.0135 (11)	0.0058 (11)	−0.0002 (11)
C22	0.0554 (17)	0.0390 (14)	0.0350 (14)	0.0135 (12)	0.0201 (12)	0.0060 (11)
C23	0.0391 (13)	0.0354 (13)	0.0445 (14)	0.0117 (10)	0.0196 (11)	0.0108 (11)
C24	0.0350 (13)	0.0337 (13)	0.0356 (13)	0.0082 (10)	0.0071 (10)	0.0066 (10)
C25	0.0220 (10)	0.0360 (13)	0.0271 (11)	0.0080 (9)	0.0058 (9)	0.0040 (10)
C26	0.0336 (12)	0.0318 (12)	0.0367 (13)	0.0095 (10)	0.0069 (10)	0.0053 (10)
C27	0.0419 (15)	0.0423 (15)	0.0397 (14)	0.0115 (11)	0.0061 (11)	−0.0066 (12)
C28	0.0493 (16)	0.0602 (18)	0.0269 (13)	0.0211 (13)	0.0095 (11)	0.0040 (12)
C29	0.0467 (15)	0.0441 (15)	0.0314 (13)	0.0148 (12)	0.0097 (11)	0.0120 (11)
C30	0.0329 (12)	0.0326 (12)	0.0326 (12)	0.0096 (10)	0.0074 (10)	0.0049 (10)
N1	0.0346 (11)	0.0441 (12)	0.0255 (10)	0.0177 (9)	0.0088 (8)	0.0089 (9)
N2	0.0285 (10)	0.0336 (10)	0.0264 (10)	0.0091 (8)	0.0059 (8)	0.0038 (8)
N3	0.0407 (12)	0.0476 (12)	0.0264 (11)	0.0233 (9)	0.0111 (9)	0.0085 (9)
N4	0.0299 (10)	0.0336 (10)	0.0275 (10)	0.0091 (8)	0.0075 (8)	0.0074 (8)

### Geometric parameters (Å, º)

**Table:**

C1—C6	1.360 (3)	C16—C17	1.380 (3)
C1—N1	1.363 (3)	C16—H16	0.9500
C1—C2	1.497 (3)	C17—C18	1.378 (3)
C2—N2	1.296 (3)	C17—H17	0.9500
C2—C3	1.433 (3)	C18—H18	0.9500
C3—C4	1.358 (3)	C19—C24	1.395 (3)
C3—H3	0.9500	C19—C20	1.397 (3)
C4—N3	1.358 (3)	C19—N3	1.409 (3)
C4—C5	1.492 (3)	C20—C21	1.368 (3)
C5—N4	1.302 (3)	C20—H20	0.9500
C5—C6	1.429 (3)	C21—C22	1.370 (3)
C6—H6	0.9500	C21—H21	0.9500
C7—C12	1.385 (3)	C22—C23	1.374 (4)
C7—C8	1.390 (3)	C22—H22	0.9500
C7—N1	1.412 (3)	C23—C24	1.381 (3)
C8—C9	1.370 (3)	C23—H23	0.9500
C8—H8	0.9500	C24—H24	0.9500
C9—C10	1.379 (3)	C25—C26	1.384 (3)
C9—H9	0.9500	C25—C30	1.402 (3)
C10—C11	1.377 (3)	C25—N4	1.409 (3)
C10—H10	0.9500	C26—C27	1.375 (3)
C11—C12	1.383 (3)	C26—H26	0.9500
C11—H11	0.9500	C27—C28	1.380 (3)
C12—H12	0.9500	C27—H27	0.9500
C13—C14	1.388 (3)	C28—C29	1.382 (4)
C13—C18	1.396 (3)	C28—H28	0.9500
C13—N2	1.412 (3)	C29—C30	1.371 (3)
C14—C15	1.374 (3)	C29—H29	0.9500
C14—H14	0.9500	C30—H30	0.9500
C15—C16	1.374 (3)	N1—H1	0.89 (3)
C15—H15	0.9500	N3—H2	0.91 (3)
			
C6—C1—N1	126.3 (2)	C18—C17—H17	119.6
C6—C1—C2	120.17 (19)	C16—C17—H17	119.6
N1—C1—C2	113.47 (17)	C17—C18—C13	120.3 (2)
N2—C2—C3	126.4 (2)	C17—C18—H18	119.8
N2—C2—C1	116.00 (19)	C13—C18—H18	119.8
C3—C2—C1	117.54 (18)	C24—C19—C20	118.5 (2)
C4—C3—C2	122.1 (2)	C24—C19—N3	119.7 (2)
C4—C3—H3	118.9	C20—C19—N3	121.8 (2)
C2—C3—H3	118.9	C21—C20—C19	120.1 (2)
N3—C4—C3	125.5 (2)	C21—C20—H20	119.9
N3—C4—C5	114.23 (18)	C19—C20—H20	119.9
C3—C4—C5	120.15 (19)	C20—C21—C22	121.1 (2)
N4—C5—C6	128.0 (2)	C20—C21—H21	119.4
N4—C5—C4	114.12 (19)	C22—C21—H21	119.4
C6—C5—C4	117.92 (18)	C21—C22—C23	119.5 (2)
C1—C6—C5	121.9 (2)	C21—C22—H22	120.2
C1—C6—H6	119.0	C23—C22—H22	120.2
C5—C6—H6	119.0	C22—C23—C24	120.5 (2)
C12—C7—C8	119.1 (2)	C22—C23—H23	119.7
C12—C7—N1	118.3 (2)	C24—C23—H23	119.7
C8—C7—N1	122.5 (2)	C23—C24—C19	120.1 (2)
C9—C8—C7	119.9 (2)	C23—C24—H24	119.9
C9—C8—H8	120.0	C19—C24—H24	119.9
C7—C8—H8	120.0	C26—C25—C30	118.5 (2)
C8—C9—C10	121.1 (2)	C26—C25—N4	124.02 (19)
C8—C9—H9	119.4	C30—C25—N4	117.3 (2)
C10—C9—H9	119.4	C27—C26—C25	120.4 (2)
C11—C10—C9	119.2 (2)	C27—C26—H26	119.8
C11—C10—H10	120.4	C25—C26—H26	119.8
C9—C10—H10	120.4	C26—C27—C28	120.9 (2)
C10—C11—C12	120.2 (2)	C26—C27—H27	119.5
C10—C11—H11	119.9	C28—C27—H27	119.5
C12—C11—H11	119.9	C27—C28—C29	119.0 (2)
C11—C12—C7	120.3 (2)	C27—C28—H28	120.5
C11—C12—H12	119.8	C29—C28—H28	120.5
C7—C12—H12	119.8	C30—C29—C28	120.6 (2)
C14—C13—C18	118.3 (2)	C30—C29—H29	119.7
C14—C13—N2	122.83 (19)	C28—C29—H29	119.7
C18—C13—N2	118.66 (19)	C29—C30—C25	120.4 (2)
C15—C14—C13	120.5 (2)	C29—C30—H30	119.8
C15—C14—H14	119.7	C25—C30—H30	119.8
C13—C14—H14	119.7	C1—N1—C7	128.76 (19)
C16—C15—C14	121.0 (2)	C1—N1—H1	111.5 (17)
C16—C15—H15	119.5	C7—N1—H1	119.7 (17)
C14—C15—H15	119.5	C2—N2—C13	121.28 (19)
C15—C16—C17	119.0 (2)	C4—N3—C19	127.73 (19)
C15—C16—H16	120.5	C4—N3—H2	110.8 (18)
C17—C16—H16	120.5	C19—N3—H2	120.5 (18)
C18—C17—C16	120.7 (2)	C5—N4—C25	123.01 (19)
			
C6—C1—C2—N2	178.4 (2)	C24—C19—C20—C21	2.0 (3)
N1—C1—C2—N2	−0.2 (3)	N3—C19—C20—C21	179.9 (2)
C6—C1—C2—C3	−3.3 (3)	C19—C20—C21—C22	−2.2 (4)
N1—C1—C2—C3	178.05 (19)	C20—C21—C22—C23	0.3 (4)
N2—C2—C3—C4	−178.1 (2)	C21—C22—C23—C24	1.7 (3)
C1—C2—C3—C4	3.8 (3)	C22—C23—C24—C19	−1.8 (3)
C2—C3—C4—N3	175.7 (2)	C20—C19—C24—C23	−0.1 (3)
C2—C3—C4—C5	−0.9 (3)	N3—C19—C24—C23	−178.0 (2)
N3—C4—C5—N4	1.4 (3)	C30—C25—C26—C27	2.0 (3)
C3—C4—C5—N4	178.4 (2)	N4—C25—C26—C27	176.7 (2)
N3—C4—C5—C6	−179.6 (2)	C25—C26—C27—C28	0.7 (4)
C3—C4—C5—C6	−2.6 (3)	C26—C27—C28—C29	−2.0 (4)
N1—C1—C6—C5	178.3 (2)	C27—C28—C29—C30	0.6 (4)
C2—C1—C6—C5	−0.1 (3)	C28—C29—C30—C25	2.1 (4)
N4—C5—C6—C1	−178.1 (2)	C26—C25—C30—C29	−3.4 (3)
C4—C5—C6—C1	3.1 (3)	N4—C25—C30—C29	−178.5 (2)
C12—C7—C8—C9	1.2 (3)	C6—C1—N1—C7	8.2 (4)
N1—C7—C8—C9	178.3 (2)	C2—C1—N1—C7	−173.3 (2)
C7—C8—C9—C10	0.6 (3)	C12—C7—N1—C1	−142.5 (2)
C8—C9—C10—C11	−1.0 (4)	C8—C7—N1—C1	40.4 (3)
C9—C10—C11—C12	−0.4 (3)	C3—C2—N2—C13	3.3 (3)
C10—C11—C12—C7	2.2 (3)	C1—C2—N2—C13	−178.59 (18)
C8—C7—C12—C11	−2.6 (3)	C14—C13—N2—C2	57.2 (3)
N1—C7—C12—C11	−179.8 (2)	C18—C13—N2—C2	−127.5 (2)
C18—C13—C14—C15	2.9 (3)	C3—C4—N3—C19	0.3 (4)
N2—C13—C14—C15	178.2 (2)	C5—C4—N3—C19	177.2 (2)
C13—C14—C15—C16	−0.8 (4)	C24—C19—N3—C4	−136.7 (2)
C14—C15—C16—C17	−1.2 (4)	C20—C19—N3—C4	45.4 (3)
C15—C16—C17—C18	1.0 (4)	C6—C5—N4—C25	5.4 (3)
C16—C17—C18—C13	1.1 (3)	C4—C5—N4—C25	−175.67 (18)
C14—C13—C18—C17	−3.0 (3)	C26—C25—N4—C5	51.4 (3)
N2—C13—C18—C17	−178.5 (2)	C30—C25—N4—C5	−133.8 (2)

### Hydrogen-bond geometry (Å, º)

Cg2 and Cg4 are the centroids of the C7–C12 and C19–C24 benzene rings, respectively.

**Table:**

*D*—H···*A*	*D*—H	H···*A*	*D*···*A*	*D*—H···*A*
C8—H8···*Cg*4^i^	0.95	2.84	3.675 (2)	148
C14—H14···*Cg*2^ii^	0.95	2.81	3.673 (3)	151

Symmetry codes: (i) −*x*+1, −*y*+1, −*z*+1; (ii) −*x*+1, −*y*, −*z*+1.
